# Gut microbiome and serum short-chain fatty acids are associated with responses to chemo- or targeted therapies in Chinese patients with lung cancer

**DOI:** 10.3389/fmicb.2023.1165360

**Published:** 2023-07-19

**Authors:** Huan-Huan Chen, Qi-Jun Wu, Tie-Ning Zhang, Yu-Hong Zhao

**Affiliations:** ^1^Department of Clinical Epidemiology, Shengjing Hospital of China Medical University, Shenyang, China; ^2^Clinical Research Center, Shengjing Hospital of China Medical University, Shenyang, China; ^3^Key Laboratory of Precision Medical Research on Major Chronic Disease, Shengjing Hospital of China Medical University, Shenyang, China; ^4^Department of Oncology, Shengjing Hospital of China Medical University, Shenyang, China; ^5^Department of Pediatrics, Shengjing Hospital of China Medical University, Shenyang, China

**Keywords:** lung cancer, gut microbiome, short-chain fatty acid, chemotherapy, targeted therapy

## Abstract

**Background:**

The association between gut microbes and short-chain fatty acids (SCFAs) and therapeutic responses of patients with lung cancer (LC) receiving therapy remains unknown.

**Methods:**

Fecal and serum samples were prospectively collected from patients with LC, classified as responders, if they presented durable clinical benefits, and non-responders, if not. The composition of gut microbes was analyzed using 16S ribosomal DNA sequencing. Serum SCFA concentrations were detected using gas chromatography. Cell proliferation, migration, invasion, cell cycle, and apoptosis assays were performed on isobutyric acid-treated A549 cells. Reverse transcription-quantitative PCR, Western blotting, immunocytochemistry, and immunofluorescence staining experiments have been performed to investigate the expression of associated genes or proteins.

**Results:**

Non-responders harbored higher microbiome α-diversity but lower β-diversity compared with responders. Compared to the patients with low α-diversity, those with high α-diversity showed significantly shorter progression-free survival. Additionally, β-diversity has also been observed between these two groups. Specifically, *Parasutterella, Clostridiaceae*, and *Prevotella_7* were more abundant among responders, whereas *Bacteroides_stercoris* and *Christensenellaceae_R-7_group* were more abundant in non-responders. The serum SCFA (especially acetate and isobutyrate) levels tended to be higher in responders. Isobutyric acid inhibited the proliferation, migration, and invasion of A549 cells by inducing apoptosis and G1/S arrest while upregulating the expression of GPR41, GPR43, and GPR5C and downregulating that of PAR1, and increasing the activity of histone acetyltransferases.

**Conclusion:**

We revealed the influence of gut microbiota and SCFAs on the therapeutic responses in patients with LC and the anti-tumor effect of isobutyric acid, indicating their potential use as therapeutic targets.

## Introduction

Lung cancer (LC) has been reported as the leading cause of death related to malignancies and as the second most common malignancy all over the world (Siegel et al., 2019). LC is a highly heterogeneous disease, including small cell LC (SCLC) and non-small cell LC (NSCLC). Despite the recent development of targeted therapies for some genetic subtypes and immune checkpoint inhibitors (ICIs) for all subtypes of human LC, the overall survival (OS) rates of patients with LC remain very low. Furthermore, the mechanisms underlying the disease incidence and progression remain poorly understood.

During the last decade, various investigations have been conducted on the association between gut microbiota and cancer. Specifically, gut microbiota were demonstrated to modulate metabolic pathways, systemic inflammation, gut barrier function, and immune cells, all of which play key roles in cancer progression (Tremaroli and Backhed, 2012; Liu et al., 2021a). Moreover, recent studies have indicated that gut microbes might be associated with the responses to therapeutic strategies against cancer. To understand the association between the microbiome and immune responses, recent studies have mainly focused on the association between microbiota and immunotherapy (Gopalakrishnan et al., 2018). For example, West and Powrie demonstrated that gut microbes might be associated with the response to immunotherapy agents of malignancies, including anti-CTLA-4 and anti-PD-L1. Mechanistically, gut microbes might strengthen the activation of dendritic and anti-tumor T-cell responses in patients with cancer (West and Powrie, 2015). Importantly, the findings reveal that immunostimulation by *Bacteroides spp*. and *Bifidobacterium spp*. had a vital effect on therapeutic response. In addition, Hakozaki et al. found that the α-diversity of the gut microbes is associated with the OS of patients with LC treated with ICIs (Hakozaki et al., 2020). *Ruminococcaceae UCG 13* and *Agathobacter* are more abundant in patients who presented a better objective response rate and progression-free survival (PFS) of greater than half a year.

Mechanistically, the gut microbiota break down dietary fibers into short-chain fatty acids (SCFAs), which could be potential mediators of the immune function of the gut microbiome. In contrast, Wang et al. illustrated that the levels of fecal SCFAs declined in patients with colorectal cancer (CRC) compared to those in healthy controls, which might be because of the depletion in SCFA-producing microbes, such as *Lachnospiraceae* and *Bifidobacterium spp*. (Wang et al., 2012). Furthermore, SCFAs act as tumor suppressors by activating G protein-coupled receptors (GPCRs) and inhibiting histone deacetylases (HDACs) (Mirzaei et al., 2021).

Despite the accumulating evidence mentioned earlier, clinical studies analyzing the potential association between gut microbes, serum SCFAs, and LC are rarely reported. To the best of our knowledge, this is the first human study exploring the combined action of gut microbes and serum SCFAs in patients with LC treated with chemotherapeutic or targeted drugs and the associated molecular mechanisms.

## Method

### Study population and sample collection

This study recruited patients diagnosed as LC between June and December 2020. Clinical information, serum, and stool samples of the included patients were collected. Details are provided in Supplementary material 1.

### 16S ribosomal DNA (rDNA) gene sequencing

The composition of gut microbiota was analyzed through rDNA sequencing. The processes include the following steps: the extraction of genome DNA, amplicon generation, quantification and quantitation of PCR products, and library preparation and sequencing. Details are provided in Supplementary material 1.

### Analysis of concentration of SCFAs

The concentration of SCFAs was quantified using gas chromatography–tandem mass spectrometry (GC-MS/MS) analysis. The materials and detailed detection methods involved are provided in Supplementary material 1, with the title of chemicals and reagents, sample preparation and extraction, and GC-MS/MS analysis.

### Cell culture and reagents

The human NSCLC cell line A549 was purchased from the Chinese Procell biological company and cultured in Ham's F-12K (PM150910; Solarbio Company, China) supplemented with 10% fetal bovine serum (FBS) in a humidified atmosphere with 5% CO_2_ at 37°C. The A549 cells were treated with 1.85 mM isobutyric acid (I103524; Aladdin Company, China) for 48 h for further experiments.

### Cell counting kit-8 assay

A CCK-8 (WLA074; Wanleibio Company, China) was used to measure cell viability. In brief, 5 × 10^3^ cells were seeded into 96-well plates in triplicate and treated with different concentrations of isobutyric acid (0.21, 0.62, 1.85, 5.56, 16.67, and 50 mM) for 48 h. CCK-8 working solution was added and allowed to react for 2 h at 37°C. Absorption at 450 nm was determined using a microplate reader (800TS; BioTek Company, USA), and the relative cell viability was calculated according to the obtained OD.

### Colony formation assay

The A549 cells were prepared in triplicate in a 60-mm Petri dish and continuously cultured for 10 d. Former colonies were fixed with 4% paraformaldehyde for 15 min and stained with Wright-Giemsa stain for 5 min. The number of colonies was counted under a light microscope, and representative images were captured using a camera.

### Transwell assay

Transwell assays were performed using transwell chambers without Matrigel (14341; LABSELECT Company, China) to evaluate cell migration and invasion. In brief, 3 × 10^4^ cells were seeded into 24-well plates in triplicate and treated with different concentrations of isobutyric acid in double distilled H_2_O (ddH_2_O), ranging between 0 and 1.85 mM. Cells in 200 μl serum-free A549 cell suspension were seeded in the upper chamber of the plate, whereas the lower chamber was filled with 800 μl Ham's F-12k supplemented with 10% FBS. After incubation for 48 h at 37°C, Transwell chambers were washed twice with phosphate-buffered saline (PBS), migrated or invaded cells were fixed with 4% paraformaldehyde for 25 min, stained with 0.4% crystal violet staining solution for 5 min, observed, and counted under a × 200 magnification microscope.

### Fluorescence-activated cell sorting analysis

For cell apoptosis, cells treated with isobutyric acid for 48 h were collected and incubated with the Annexin V-FITC Apoptosis Detection Kit (WLA001; Wanleibio Company, China) for 15 min at 25°C. FACS analysis was performed using propidium iodide staining. For cell cycle analysis, cells treated with isobutyric acid for 48 h were fixed with 70% ethanol and incubated with the A-PI Cell Cycle Detection Kit (WLA010; Wanleibio Company, China) for 30 min at 25°C according to the manufacturer's instructions. After incubation, approximately 5 × 10^5^ cells were analyzed using a flow cytometer (ACEA Company, NovoCyte, USA).

### Reverse transcription-quantitative PCR

Total RNA was isolated from the indicated cell lines using TriPure lysate (RP1001; BioTek, China). RNA aliquots (1 μg) were then reverse-transcribed using the Exicycler^TM^ 96 Real-Time PCR instrument (BIONEER Company, Korea) according to standard protocols: 10 min at 25°C, 50 min at 42°C, and 10 min at 80°C. qRT-PCR was performed using the Exicycler^TM^ 96 Real-Time PCR instrument according to the manufacturer's instructions. qPCR was performed on the reverse-transcribed cDNA samples using the SYBR Green qPCR Master Mix (SY1020; Solarbio Company, China). Thermocycling conditions were as follows: 94°C for 5 min, followed by 40 cycles at 94°C for 10 s, 60°C for 20 s, and 72°C for 30 s. The fluorescence threshold value was calculated using the 2^−ΔΔCq^ method (PCR primers see Supplementary Table 1).

### Western blot analysis

Cells were washed once with PBS and lysed in lysis buffer on ice for 5 min. Cell lysates were centrifuged at 12,000 rpm for 10 min at 4°C, boiled in 5 × loading buffer, and subjected to protein determination (BSA, WLA004; Wanleibio Company, China). Equal amounts (15–30 μg/well) of protein samples were separated using sodium dodecyl sulfate–polyacrylamide gel electrophoresis and transferred to polyvinylidene difluoride membranes. Membranes were first blocked with 5% non-fat dry milk and incubated with primary antibodies specific against acetyl-histone H3, acetyl-histone H4, GPR41, GPR43, GPRC5A, and PAR1 at a 1:1,000 dilution ratio and against histone H3 and histone H4 at a 1:500 dilution ratio. Samples were probed with anti-acetyl-histone H3 antibodies (A7255; Abclonal Company, China), anti-histone H3 antibodies (WL0984a; Wanleibio Company, China), anti-acetyl-histone H4 antibodies (A7258; Abclonal Company, China), anti-histone H4 antibodies (A1131; Wanleibio Company, China), anti-GPR41 antibodies (66811-1-Ig; Proteintech Company, China), anti-GPR43 antibodies (DF2746; Affinity Company, China), anti-GPRC5A antibodies (DF5148; Affinity Company), and anti-PAR1 (AF0263; Affinity Company) at 4°C overnight. Membranes were then stained with secondary antibodies at 1:5,000 dilution at 25°C for 45 min, and an ECL solution (WLA003; Wanleibio Company, China) was used for visualization. The results were analyzed using the Gel-Pro Analyzer software (version 4.0; Media Cybernetics, USA).

### Immunocytochemical staining

A549 cells were seeded in triplicate into 12-well plates and fixed with 4% paraformaldehyde for 15 min. Subsequently, cells were incubated with 0.1% Triton X-100 (1 μl Triton X-100 dissolved in 1 ml PBS; ST975, Beyotime Company, China). Endogenous peroxidase activity was blocked using 3% hydrogen peroxide for 15 min. PBS was removed from the 12-well plates, and cells were incubated with 1% BSA for 15 min at 25°C. Anti-acetyl-histone H3, anti-GPR41, anti-GPR43, anti-GPRC5A, and anti-PAR1 antibodies were applied at a dilution of 1:100, whereas anti-acetyl-histone H4 antibody was applied at a dilution of 1:500, and all samples were incubated at 4°C overnight. The signal was visualized using diaminobenzidine. Sections were counterstained with hematoxylin. ICC results were scored by accounting for the percentage of positive detection and intensity of staining. The intensity score was as follows: 0, no staining; 1, weakly positive; 2, moderately positive; and 3 and 4, strongly positive.

### Immunofluorescence staining

A549 cells were fixed with 4% (w/v) paraformaldehyde for 15 min at 25°C and washed twice with PBS. Cells were then incubated in 0.1% (v/v) Triton X-100 in PBS for 30 min and then blocked with 1% BSA in PBS for 15 min at 25°C. After blocking, cells were incubated with anti-acetyl-histone H3, anti-GPR41, anti-GPR43, anti-GPRC5A, and anti-PAR1 antibodies at a dilution of 1:100, whereas anti-acetyl-histone H4 antibody was applied at a dilution of 1:500. All samples were incubated at 4°C overnight, then washed twice with PBS and incubated for a further 1 h with a secondary antibody (conjugated to fluorescent dye; 1:200 dilution), and finally washed thrice with PBS. After completing all necessary washing steps, cell nuclei were counterstained with 4′,6-diamidino-2-phenylindole (D106471-5 mg; Aladdin Company, China). Coverslips were inverted onto a glass slip with 20–40 μl fluorescent mounting medium (S2100; Aladdin Company), and cells were observed under a fluorescence microscope.

### Histone acetyltransferase activity assay

Nuclear protein extraction was performed using the Nuclear and Cytoplasmic Protein Extraction Kit (WLA020; Wanleibio Company, China) according to the manufacturer's instructions. Proteins were quantified using a BCA Protein Quantification Kit (WLA004; Wanleibio Company, China) according to the manufacturer's instructions. Protein samples (50 μg) were subjected to the HAT activity assay (ab65352; Abcam Company, China) as follows: (1) Preparation of test samples: 50 μg nuclear protein was dissolved in 40 μl water, with 40 μl ddH_2_O being added as background control. (2) Assay mix: 68 μl assay mix (50 μl 2 × HAT detection buffer, 5 μl HAT substrate I, 5 μl HAT substrate II, and 8 μl NADH generating enzyme) was prepared and added to each well. (3) The plate was incubated at 37°C for 2 h. Absorption was determined at 440 nm. We determined HAT activity (OD/μg protein) as follows:


$$\begin{array}{l} \left(OD\;sample-OD\;background\right)/50\;\mu gprotein \end{array}$$

### Statistical analysis

The statistical methods for analyzing the data of gut microbiota, SCFA, and *in vitro* experiments are provided in Supplementary material 1, with the title of statistical analysis of gut microbiota data, statistical analysis of SCFA levels, Spearman's correlation analysis, and statistical analysis of *in vitro* experiments.

## Results

### Baseline characteristics of the population

In total, we collected stool and serum samples from 60 patients with LC. Among them, 30 patients are defined as responders, whereas 30 patients are defined as non-responders. There are no significant differences in age, sex, disease stage, smoking history, pathological type, and Eastern Cooperative Oncology Group scores, and treatment between responders and non-responders was observed (Table 1).

**Table 1:** Clinical characteristics of participants.

**Baseline characteristic**	**Responder (*n =* 30)**	**Non-responder (*n =* 30)**	***p-*value**
**Age (year), mean** **±SD**	61.13 ± 4.96	62.13 ± 6.29	0.436
≥65, *n* (%)	7 (23.3%)	14 (46.7%)	
< 65, *n* (%)	23 (76.7%)	16 (53.3%)	
**Sex**, ***n*** **(%)**			0.781
Male	21 (70%)	20 (66.7%)	
Female	9 (30%)	10 (33.3%)	
**Clinical stage**, ***n*** **(%)**			0.828
I	2 (6.7%)	1 (3.3%)	
II	0	0	
III	17 (56.7%)	17 (56.7%)	
IV	11 (36.7%)	12 (40%)	
**Smoking history**, ***n*** **(%)**			0.796
Yes	15 (50%)	16 (53.3%)	
No	15 (50%)	14 (46.7%)	
**Pathological type**, ***n*** **(%)**			0.666
SCLC	10 (33.3%)	11 (36.7%)	
NSCLC			
Squamous cell carcinoma	9 (30%)	6 (20%)	
Adenocarcinoma	11 (36.7%)	13 (43.3%)	
**ECOG score**, ***n*** **(%)**			0.554
0	1 (3.3%)	2 (6.7%)	
1	29 (96.7%)	28 (93.3%)	
**Treatment**			0.312
Chemotherapy	26	22	
Targeted therapy	4	8	

SD, standard deviation; ECOG, Eastern Cooperative Oncology Group.

### Differences in α- and β-diversity between responders and non-responders

**Figure 1:**
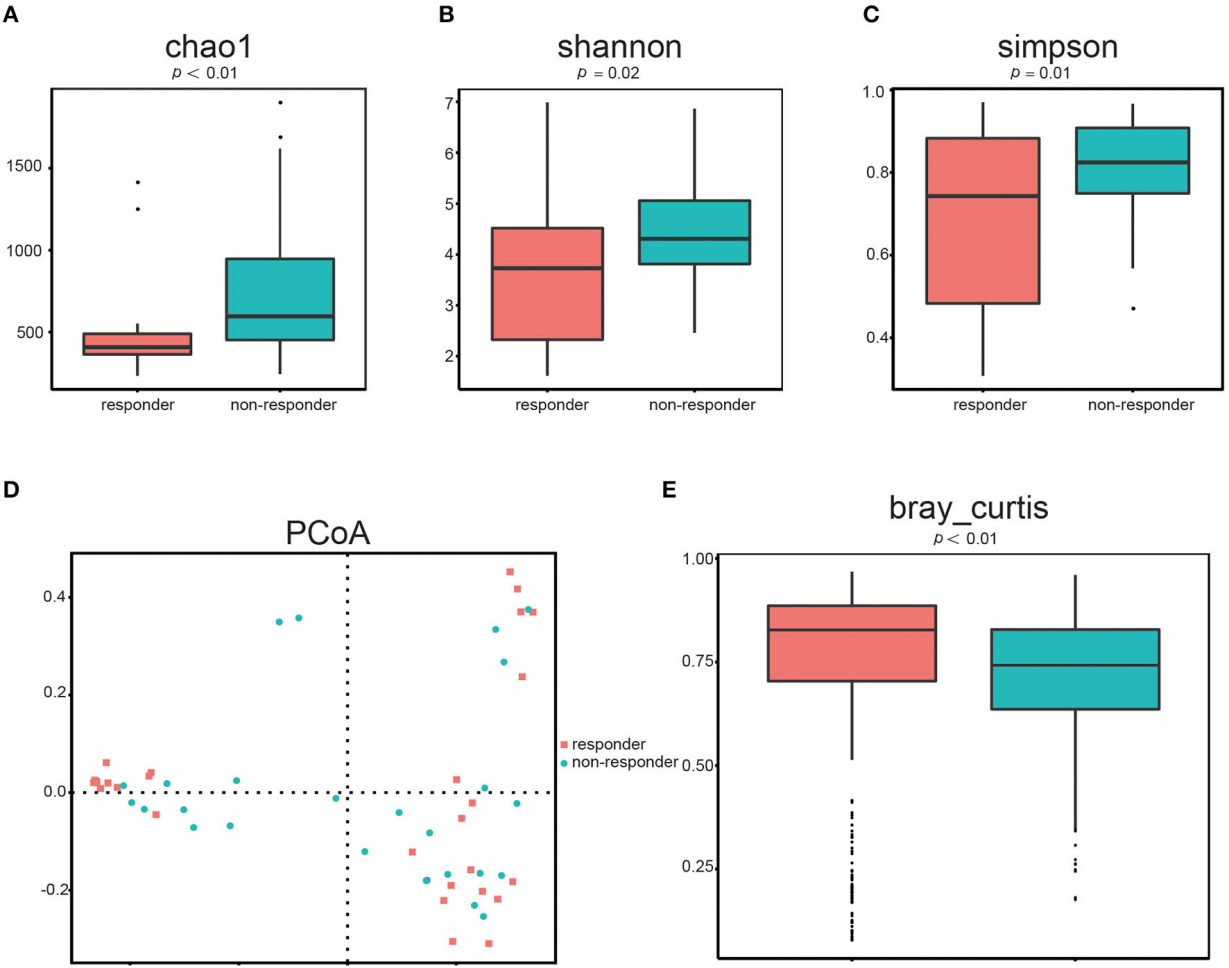
Gut microbial diversity in responders (*n* = 26) and non-responders (*n* = 28). α-diversity was evaluated based on the Chao1 **(A)**, Shannon **(B)**, and Simpson **(C)** indices of the OTU levels. Principal coordinate analysis of β-diversity was based on the PCoA **(D)** and Bray–Curtis **(E)** analyses of the OTU levels.

First, we determined the microbial diversity using the Chao1, Shannon, and Simpson indices. As a result, we found that α-diversity was significantly reduced in responders compared to that in non-responders (Figure 1A: Chao1, *p* < 0.01, Figure 1B: Shannon, *p* = 0.02, Figure 1C: Simpson, *p* = 0.01). These results indicated that the intraindividual bacterial diversity in responders was distinctly different from that in non-responders.

Second, we used the PCoA (Figure 1D) of Bray–Curtis to quantify β-diversity in two groups. The Bray–Curtis distances demonstrated that gut microbes in responders are significantly different from that in non-responders (Figure 1E; *p* < 0.01), further indicating that β-diversity differed among the two groups. Hence, the structural diversity of the gut microbiota might be closely related to its therapeutic effect in patients with LC.

### Distinct composition of gut microbes between the two groups

**Figure 2:**
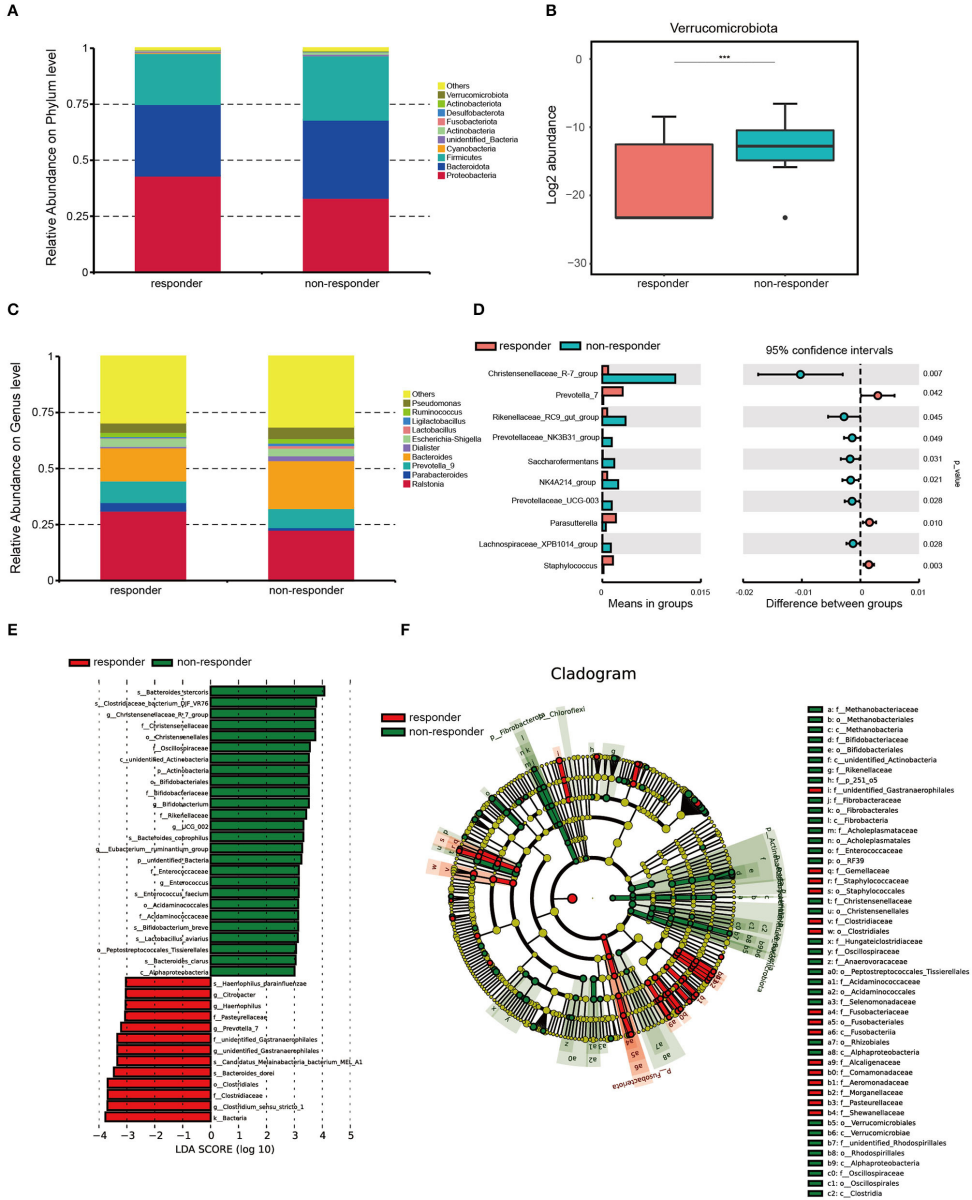
Microbiota composition in responders (*n* = 26) and non-responders (*n* = 28). **(A)** Summary of the relative abundances of phyla present in the study population. **(B)** Log2 abundance of Verrucomicrobiota. Verrucomicrobiota were significantly elevated in non-responders compared with responders. Statistically significant differences according to the MetaStat are marked with asterisks. ***p < 0.001, **p < 0.01, and *p < 0.05. **(C)** Summary of the relative abundances of bacterial genera detected in responders and non-responders. **(D)** Genus-level bacteria that were significantly different between the responders and non-responders. Statistical analysis was performed by the *t*-test. **(E)** Histogram of LDA scores to identify differential abundant bacteria between responders and non-responders (LDA score > 3). **(F)** Cladogram generated from the LEfSe analysis indicating the phylogenetic distribution from class to species of the gut microbiota of responders and non-responders.

We found that *Proteobacteria* was the most enrichment phylum in both groups, and the next are *Bacteroidetes, Firmicutes, Cyanobacteria, unknown_Bacteria, Actinobacteria, Fusobacteriota, Desulfobacterota*, and *Verrucomicrobiota* (Figure 2A). Among them, *Verrucomicrobiota* was significantly more abundant in non-responders than in responders (*p* < 0.001; Figure 2B). The individual data of taxonomic composition distribution in responders and non-responders on the phylum level are shown in Supplementary Figure 1A.

We also performed MetaStat to compare the differences in the composition of fecal bacterial communities at the genus level between the two groups (Figure 2C). We found that the abundances of *Christensenellaceae_R-7_group* (*p* < 0.01), *Prevotella_7* (*p* = 0.04), *Rikenellaceae_RC9_gut_group* (*p* = 0.04), *Prevotellaceae_NK3B31_group* (*p* = 0.05), *Saccharofementans* (*p* = 0.03), *NK4A214_group* (*p* = 0.02), *Prevotellaceae_UCG-003* (*p* = 0.03), *Parasutterella* (*p* = 0.01), *Lachnosiraceae_XPB1014_group* (*p* = 0.03), and *Staphylococcus* (*p* < 0.01) are significantly different between the two groups (Figure 2D). The individual data of taxonomic composition distribution in responders and non-responders on genus level are shown in Supplementary Figure 1B.

We used LEfSe to further analyze whether specific bacterial taxa showed different abundances between the two groups. By using the logarithmic LDA score cutoff of 3, 39 key discriminants are identified (see Supplementary Table 2 and Figure 2E). Specifically, we identified one kingdom, *Bacteria* (*p* = 0.009), one genus, *Clostridium_sensu_stricto_1* (*p* = 0.014), and one family, *Clostridiaceae* (*p* = 0.025), that were significantly overrepresented in the feces of responders. Conversely, two species, *Bacteroides_stercoris* (*p* = 0.004) and *Clostridlaceae_bacterium_DJF_VR76* (*p* < 0.001), and one genus, *Christensenellaceae_R-7_group* (*p* < 0.001), were enriched in non-responders. Interestingly, a cladogram of the taxonomic hierarchical structure of gut microbes from class to species shows significant differences in the phylogenetic distributions between the microbes of the two groups (Figure 2F). As a result, there are notable differences in the composition of gut microbes between the two groups.

### Higher levels of serum acetate and isobutyrate in responders

**Figure 3:**
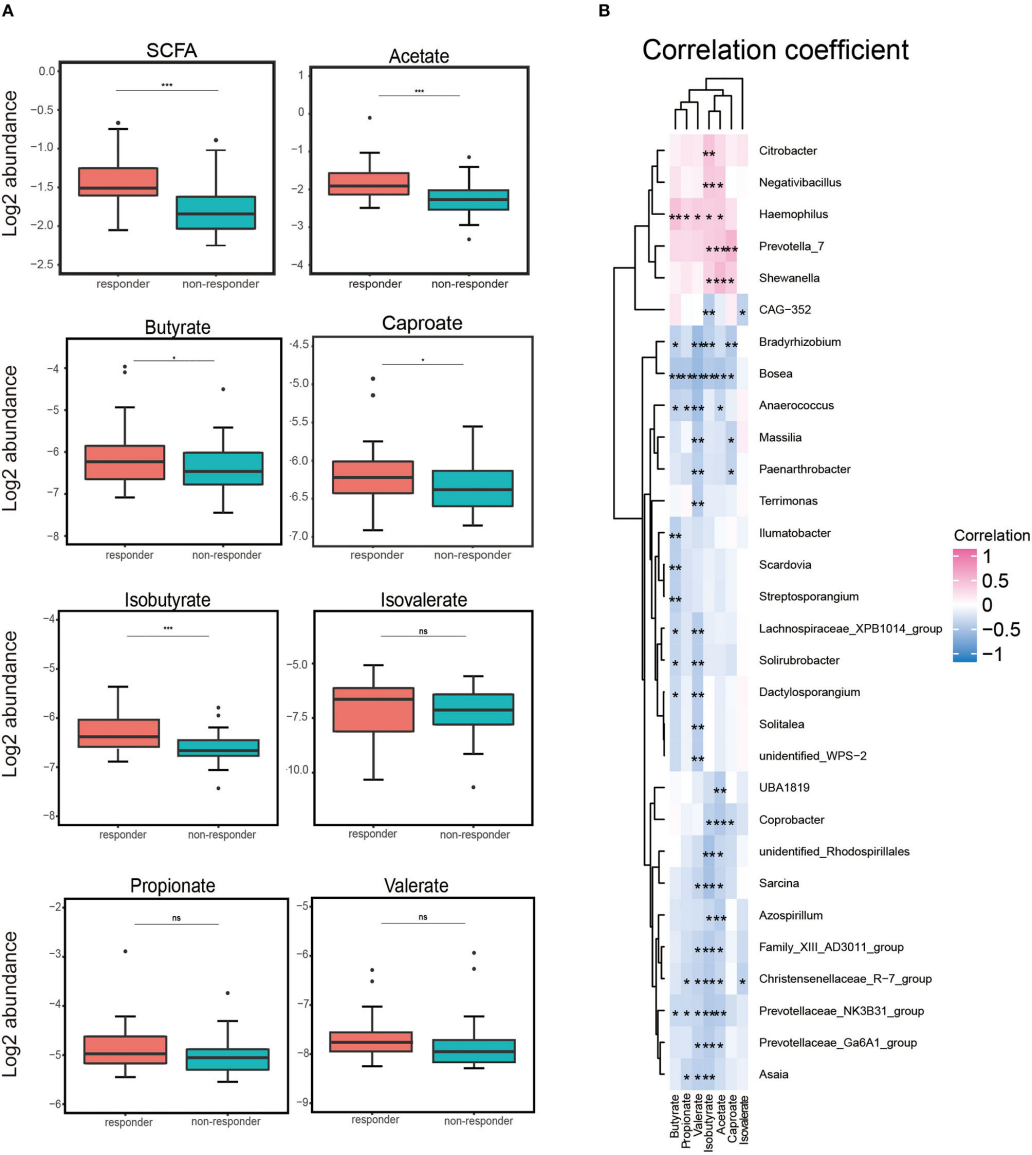
Log2 abundance of short-chain fatty acids (SCFAs) and Spearman's analysis between gut microbiota and SCFA. **(A)** Log2 abundance of total SCFA and acetate, butyrate, caproate, isobutyrate, isovalerate, propionate, and valerate separately. Log2 abundance of acetate and isobutyrate was found to be reduced significantly in non-responders (*n* = 30) compared with responders (*n* = 30). *P*-values from the *t*-test are shown. ***p < 0.001, **p < 0.01, and *p < 0.05. **(B)** Spearman's correlation analysis between the gut bacteria genus and serum SCFA. Positive and negative correlations are shown as pink and blue in the heat map, respectively. Significant microbiota–metabolite correlations were determined based on an | r | ≥ 0.8 and *p* < 0.05 (*p < 0.05).

We assessed the serum levels of SCFAs (acetate, butyrate, caproate, isobutyrate, isovalerate, propionate, and valerate) in a subgroup of 60 participants (30 responders and 30 non-responders). We detected that the total serum concentrations of SCFAs tended to be higher in responders compared to those in non-responders. We also observed the same pattern that the level in the responders tended to be higher when analyzing each SCFA separately (see Supplementary Figure 2). The log2 abundance of total SCFA, acetate, and isobutyrate was remarkably higher in responders than in non-responders (*p* < 0.001). We found that levels of butyrate and caproate were notably higher in responders (*p* < 0.05). Although serum concentrations of propionate, valerate, and isovalerate were consistent, the difference was not statistically significant (Figure 3A).

### Correlations between gut microbiota and short-chain fatty acid

We used the Spearman correlation coefficient to calculate the coefficients of different genera and serum SCFAs and explore the correlation between an imbalance in the composition of gut microbes and alterations in serum SCFA concentrations. As shown in Figure 3B, it was worth mentioning that we found that *Haemophilus* was positively correlated with five SCFA levels (*p* < 0.01: acetate, propionate, valerate, and isobutyrate; *p* < 0.001: butyrate), potentially indicating a strongly synergistic effect between *Haemophilus* and SCFAs, especially butyrate. Conversely, *Bosea, Christensenellaceae_R*−*7_group*, and *Prevotellaceae_NK3B31_group* were negatively correlated with at least five SCFA levels. Among them, we observed a negative correlation between *Bosea* and six SCFA levels (*p* < 0.01: caproate; *p* < 0.001: acetate, butyrate, propionate, valerate, and isobutyrate). This negative correlation might indicate an antagonistic effect between these microorganisms and their metabolites, especially *Bosea* and serum SCFA concentrations. These data suggested that patients with LC exhibit notable taxonomic disturbances in the gut microbes, potentially leading to a markedly altered SCFAs' profile.

### Isobutyrate suppressed the malignant biological properties of LC cells

**Figure 4:**
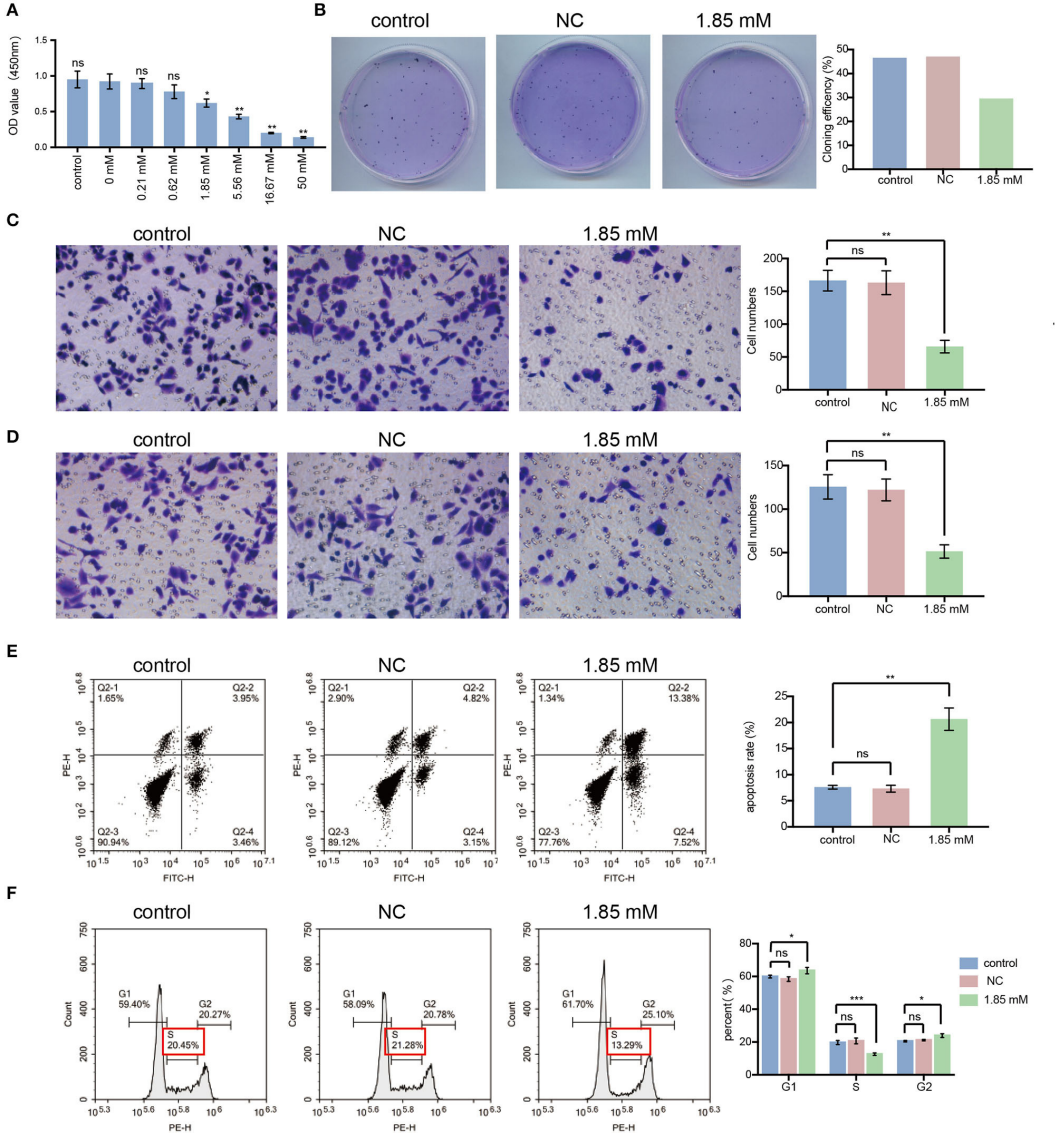
Isobutyric acid suppresses the malignant cellular biological behavior, induces cell apoptosis and G1/S arrest in lung cancer cells. **(A)** After isobutyric acid treatment (control, NC, 0.21 mM, 0.62 mM, 1.85 mM, 5.56 mM, 16.67 mM, and 50 mM), the cell proliferation activity decreased gradually with the increase of isobutyric acid concentration. **(B)** After isobutyric acid treatment (control, NC and 1.85 mM), A549 cells were fixed in 4% paraformaldehyde and stained with Wright-Giemsa Stain. The cell cloning efficiency decreased in the 1.85 mM treatment group compared with the control groups. After isobutyric acid treatment (control, NC and 1.85 mM), A549 cells were fixed in 4% paraformaldehyde and stained with 0.4% crystal violet. Both the numbers of cell migration **(C)** and invasion **(D)** decreased in the 1.85 mM treatment group compared with the control groups. The cells were imaged with a × 200 magnification microscope. FACS analysis using propidium iodide staining was performed after treatment of A549 cells with isobutyric acid (control, NC and 1.85 mM). **(E)** Both early and late phase apoptosis rate of A549 cells increased in the 1.85 mM treatment group compared with the control groups. **(F)** After isobutyric acid treatment, the percentage of A549 cells increased in the S phase while decreased in the G1 phase in the 1.85 mM treatment group compared with the control groups (NC and 1.85 mM). *P-*values were calculated using Student's *t*-tests. ns: no significance; *p < 0.05; **p < 0.01; and ***p < 0.001. All experiments were repeated three times. NC, negative control (0 mM).

We found that serum acetate and isobutyrate levels were markedly reduced in non-responders. Thus, we assumed that acetate and isobutyrate might act as tumor suppressors in LC. Previous studies explored the molecular mechanism of the anti-cancer effect of acetate (Marques et al., 2013; Xia et al., 2016), but there is little evidence about the influence of isobutyrate on the biological characteristics of cancer cells, especially LC. To explore the tumor suppressor role of isobutyrate in LC, we performed cell proliferation, migration, and invasion assays on A549 cells after treatment with isobutyric acid. Compared with the control group, we noticed that cell activity was remarkably decreased in the isobutyric acid-treated group. The inhibitory effect of isobutyric acid on the activity of LC cells was concentration-dependent, exhibiting statistical significance at concentrations of 1.85 mM (*p* < 0.05), 5.56 mM (*p* < 0.01), 16.67 mM (*p* < 0.01), and 50 mM (*p* < 0.01) (Figure 4A). Thus, we chose the minimum effective concentration (1.85 mM) for subsequent experiments. After treatment with 1.85 mM isobutyric acid, we found that the cell cloning rate (29.50%) was lower than that in the control (46.50% for the control and 47% for the negative control, NC, 0 mM; Figure 4B).

As shown in Figures 4C, D, isobutyric acid prevented the migration and invasion of A549 cells. Statistically, the average number of migrating and invading isobutyric acid-treated A549 cells was < 65 and 50, respectively, which was a significant reduction compared with the respective number of cells (more than 165 migrating cells, *p* < 0.01, and more than 125 invading cells, *p* < 0.01) in control groups. Hence, we propose an alternative therapeutic method using isobutyric acid for treating LC.

### Isobutyrate-induced G1/S arrest and cell apoptosis in A549 cells

We assessed whether the inhibition of isobutyric acid on the malignant biological properties of A549 cells was related to the influences of isobutyric acid on apoptosis and the cell cycle of LC cells. We performed FACS analysis to analyze the influence of isobutyric acid on apoptosis and cell cycle in A549 cells. As shown in Figure 4E, the proportion of cells in the early and late phases of apoptosis in the isobutyric acid-treated group was higher than that in the control group (*p* < 0.01). Moreover, we observed G1/S arrest in A549 cells after treatment with isobutyric acid (Figure 4F), implying that isobutyric acid-induced G1/S arrest and apoptosis, thus inhibiting the growth of LC cells.

### Isobutyrate regulated the expression of GPCRs and activated HAT

**Figure 5:**
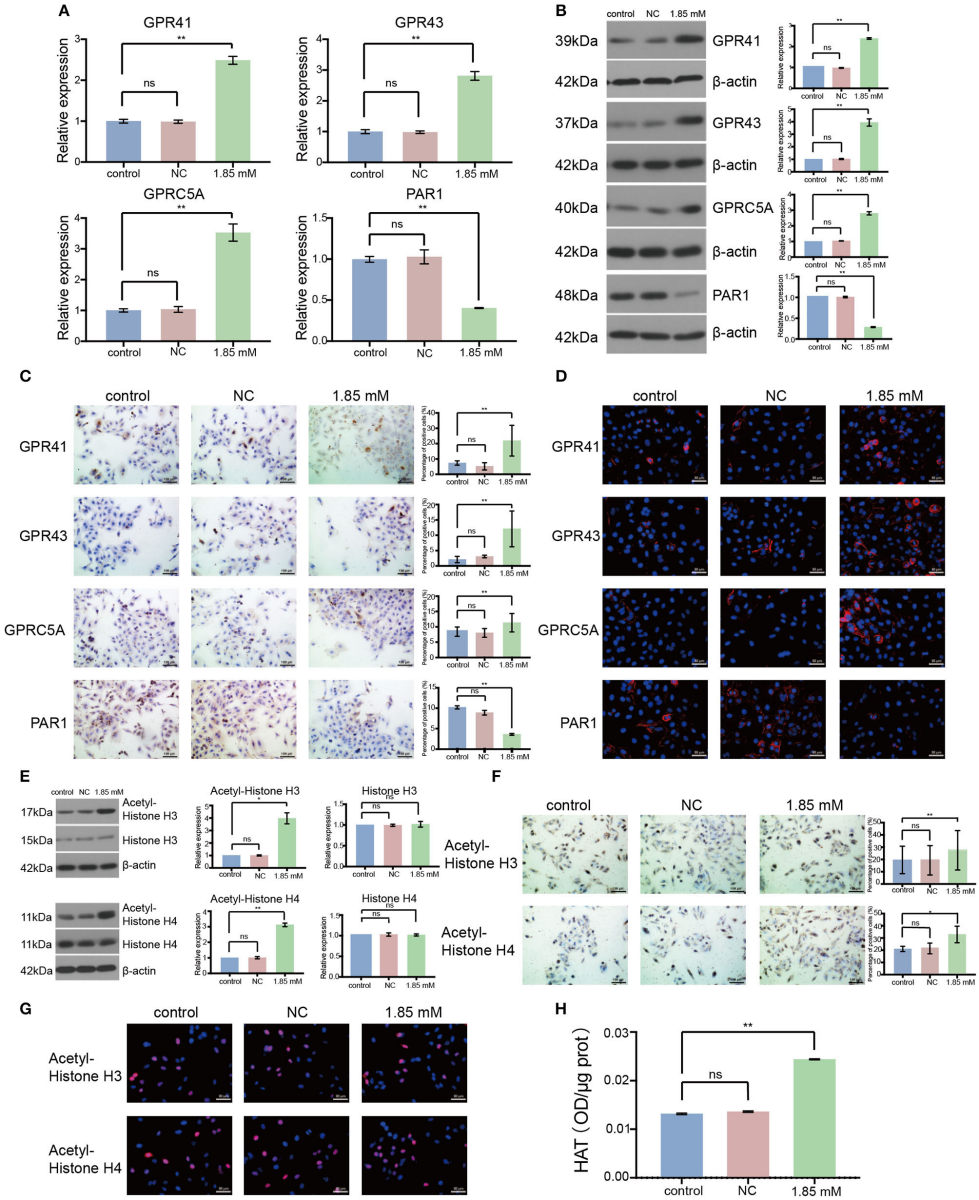
Isobutyric acid regulated G protein-coupled receptor (GPCR) expression and histone acetyltransferase (HAT) activity. For the expression of GPCRs, the results of RT-qPCR **(A)**, Western blot **(B)**, and immunocytochemical staining **(C)** showed that GPR41, GPR43, and GPRC5A expressions were significantly higher, while PAR1 expression was significantly lower in isobutyric acid treatment group (1.85 mM) than the control groups (control and NC). Immunofluorescence staining **(D)** identified the expression location of GPCRs. For the expression of acetyl-histones and histones, the results of Western blot **(E)** and immunocytochemical staining **(F)** showed that acetyl-histone H3 and H4 expressions were significantly higher in the isobutyric acid treatment group (1.85 mM) than the control groups (control and NC), whereas Western blot results showed that there was no significant difference of the expression of histone H3 and H4 between isobutyric acid treatment group and the control groups. Immunofluorescence staining (G) identified the expression location of acetyl-histone H3 and H4. HAT activity assay **(H)** results showed that HAT activity was increased in the isobutyric acid treatment group (1.85 mM) than in the control groups (control and NC). P-values were calculated using Student's *t-*tests. ns: no significance; *p < 0.05; **p < 0.01; ***p < 0.001. All experiments were repeated three times. NC, negative control (0 mM).

To analyze the role of isobutyric acid at the molecular level, we designed primers to amplify GPCR. As a result, we found that the expression levels of GPR41, GPR43, and GPRC5A were significantly increased, whereas that of PAR1 significantly decreased after treatment of A549 cells with isobutyric acid (Figure 5A: *p* < 0.01). Moreover, we detected that the expression of GPCRs was clearly induced by the treatment of LC cells with isobutyric acid, as observed by Western blotting (Figure 5B: *p* < 0.01). Furthermore, GPCRs were notably induced or reduced in the isobutyric acid-treated cells compared to that in the controls (Figure 5C: *p* < 0.01). Additionally, we analyzed the location of the expression of GPCRs using immunofluorescence staining (Figure 5D). Our results revealed that treatment with isobutyric acid affected the transcription of GPCR-related genes, inhibiting the growth of LC cells.

We performed similar experiments to detect the expression of acetylated histones and histones in A549 cells. We found that the expression of acetylated H3 and H4 histones was remarkably higher in the isobutyric acid-treated group than that in the control group (Figure 5E: acetylated histone H3, *p* < 0.05, acetylated histone H4, *p* < 0.01; Figure 5F: acetylated histone H3, *p* < 0.01; acetylated histone H4, *p* < 0.05). Furthermore, we analyzed the localization of the expression of acetylated histones using immunofluorescence staining (Figure 5G). However, we did not detect any significant difference in the expressions of H3 and H4 histones between the isobutyric acid-treated and control groups (Figure 5E). We found that isobutyric acid upregulated the levels of acetylated histones by activating HAT. Next, we analyzed the activity of HAT in LC cells after treatment with isobutyric acid using the Histone Acetyltransferase Activity Assay. We found that the activity of HAT was significantly higher in A549 cells treated with isobutyric acid than that in the controls (Figure 5H: *p* < 0.01). In summary, the results showed that being treated with isobutyric acid increased apoptosis and induced cell cycle arrest through regulating the expression of GPCRs and activity of HAT in LC cells; these data provided useful information on the potential anti-cancer role of isobutyric acid for the treatment of LC.

## Discussion

This is the first study to explore the composition of gut microbes, concentrations of SCFAs, and their association with responses to chemotherapy or targeted therapy in patients with LC. Both the α-diversity and β-diversity were different between responders and non-responders. Based on previous studies showing the association of reduced diversity with an unfavorable response to immunotherapy (Jin Y. et al., 2019; Heshiki et al., 2020), we expected to detect reduced α- and β-diversities in patients with LC who responded worse to chemotherapy or targeted therapy (non-responders). Moreover, higher microbial diversity usually associates with better health, whereas reduced microbial diversity usually associates with several disease states (Lozupone et al., 2012). Despite identifying reduced β-diversity in non-responders, α-diversity was higher in non-responders in our study population. However, various factors influence the biodiversity of human gut microbiota, including antibiotic application (Becattini et al., 2016), dietary habits (Rinninella et al., 2019), obesity (Gerard, 2016), and environmental factors (Anwar et al., 2021). Hence, more attention should be paid to the key microorganisms that were notably different between the two groups.

In addition, differential abundances of certain bacterial taxa have been found between responders and non-responders. Specifically, *Parasutterella* was found to be more abundant among responders in our study. Pi et al. reported an elevated abundance of *Parasutterella* in patients with CRC after anti-PD-1 treatment (Pi et al., 2020). Additionally, Messaoudene et al. observed that camu-camu reversed the anti-PD-1 resistance through direct interactions with commensal bacteria. In animal models, the numbers of *Parasutterella* increased after treatment with camu-camu and anti-PD-1 (Messaoudene et al., 2022). In this study, the higher abundance of *Clostridiaceae* and *Prevotella_7* seemed to be related to a better response to chemotherapy or targeted therapy in patients with LC. Although the role of these bacteria in anti-tumor therapeutic efficacy has seldom been reported, their association with cancer risk is known from previous studies. For example, one network analysis reported that a higher abundance of *Clostridiaceae* was closely related to a lower risk of NSCLC (Vernocchi et al., 2020). However, an elevated abundance of *Prevotella_7* appeared to increase the risk of cancer. Liu et al. found that the abundance of *Prevotella_7* was higher in patients with choriocarcinoma than in those with invasive moles (Liu et al., 2021b). Moreover, receiver operating characteristic analysis showed that *Prevotella_7* might be a potential biomarker for the identification between choriocarcinoma and invasive moles. A similar result was reported by Wu et al., revealing that the higher relative abundance of *Prevotella_7* was related to the increased risk of gastric cancer (Wu et al., 2020).

Conversely, *Bacteroides_stercoris* was significantly more abundant among non-responders. Andrews et al. reported a contrasting result while studying the association between gut microbiota and the efficacy of anti-CTLA-4 combined with anti-PD-1 in melanoma patients (Andrews et al., 2021). Andrews et al. found that *Bacteroides_stercoris* was more abundant in patients with melanoma who exhibited longer PFS. Moreover, the *Christensenellaceae_R-7_group* was also found to be more abundant in non-responders. One meta-analysis showed that the *Christensenellaceae_R-7_group* was more abundant in patients with gut diseases, including CRC, compared with healthy controls (Mancabelli et al., 2017). However, an association between the *Christensenellaceae_R-7_group* and anti-tumor drug response has seldom been reported. Finally, although the results of our study are not entirely consistent with those of previous studies on other cancer types, we assumed that *Parasutterella* and *Bacteroides_stercoris* might be biomarkers for predicting therapeutic efficacy in patients with NSCLC. Furthermore, elucidating the role of markedly different bacteria between the two groups in anti-cancer drug response might be a direction for future research.

In addition, this study explored the association between the serum concentration of SCFAs and chemotherapeutic or targeted drug efficacy in LC. The serum SCFA (especially acetate and isobutyrate) levels in responders were higher than those in non-responders. SCFAs show anti-inflammatory effects through interacting with the immune system, thus improving gut barrier integrity in patients with malignancies (Martinez et al., 2012; Bach Knudsen et al., 2018). Notably, the destruction of gut barrier integrity promotes and contributes to cancer progression (Martinez et al., 2012). In addition, it was recently shown that following anti-PD1 treatment, the levels of acetate, butyrate, and propionate decreased in non-responders compared to those in the responders with various solid cancers, including melanoma, head and neck cancer, gastrointestinal cancer, genitourinary cancer, lung adenocarcinoma, and sarcoma (Yu et al., 2011; Nomura et al., 2020). This finding indicated that the fecal and plasma concentrations of SCFAs were associated with the efficacy of anti-PD-1 treatment. In our study, we speculated that the altered levels of acetate and isobutyrate were associated with the anti-inflammatory effects in cancer tissues and closely related to the response of patients to drugs.

SCFAs are major end-product metabolites of the gut microbiota, with several bacterial producers, such as *Prevotella, Bifidobacterium*, and *Bacteroides*, being confirmed in previous studies (Yu et al., 2011; Murugesan et al., 2018). Accordingly, we performed a combined analysis of the gut microbiota and serum SCFA levels using Spearman's rank correlation coefficient. We observed a significant positive or negative correlation between SCFAs and some genera. Moreover, WGCNA by Vernocchi et al. generated strong correlations between the SCFA levels and a healthy gut microbiota by comparing PD1-treated patients with NSCLC to the healthy controls (Vernocchi et al., 2020). The association between SCFAs and gut microbiota illustrated that the identification of gut microbe-related biomarkers might indicate personalized treatment for patients with LC.

At the molecular level, SCFAs derived from the microbiome inhibit the growth of various cancers, such as colon and lung cancer, by modulating GPCRs, histone hyperacetylation, or through the dysregulation of the expression of BCL-2 and PCNA (Zhao et al., 2018; Kim et al., 2019; Mirzaei et al., 2021). However, studies on SCFAs focus on acetate, butyrate, and propionate, and the effect of isobutyrate in LC is not clear. Therefore, we further investigated the effect of isobutyric acid on A549 cells and demonstrated novel findings regarding its role in the treatment of LC. Isobutyric acid inhibited the malignant biological properties of LC cells by inducing G1/S phase arrest and increasing apoptosis. In addition, we found that isobutyric acid regulated the expression of GPCRs and the activity of HAT in the A549 LC cell line. The upregulation of GPR41, GPR43, and GPRC5A expression, as well as the activity of HAT and the downregulation of PAR1 expression, have previously been shown to be mainly involved in cell cycle arrest (Miao et al., 2010; Tang et al., 2011; Wu et al., 2012; Sawada et al., 2020; Ribelli et al., 2021). Moreover, SCFAs regulated the expression of GPCRs and subsequently affected cancer progression, as confirmed in previous studies. For example, propionate inhibited colon cancer progression by upregulating the expression of GPR43 and GPR109A (Tang et al., 2011; Singh et al., 2014). The interaction between GPRC5A or PAR1 and SCFAs, especially isobutyrate, was reported for the first time in this study. Interestingly, GPRC5A might have contrasting roles in different solid malignant tumors, such as facilitating cell proliferation in prostate cancer (Sawada et al., 2020), whereas suppressing cell viability and inducing cell apoptosis in LC (Jin et al., 2017). Furthermore, Jin et al. found that the low expression of GPRC5A indicated a worse prognosis in NSCLC (Jin E. et al., 2019). In addition, PAR1 has been found to have contrasting functions in various cancer types (Liu et al., 2017; Adams et al., 2018). For instance, Adams et al. identified that PAR1 impeded prostate and intestinal tumor progression in mice (Adams et al., 2018). However, in LC, the proliferation of cancer cells notably reduced when the expression of PAR1 was knocked down (Wu et al., 2014).

Studies showed that SCFAs regulated the levels of acetylated histones by inhibiting HDACs. More specifically, they showed that butyrate bounds to HDACs and acts as an HDAC inhibitor in colon cancer (Wu et al., 2018). Surprisingly, we found that isobutyric acid activated HAT in A549 cells. An *in vitro* study showed that mesoderm induction early response 1, family member 3 (MIER3) inhibited the proliferation of A549 and H460 cells by decreasing the activity of HAT p300 (Zhang et al., 2020). A study performed by Liu et al. found that Snail transcription was repressed, whereas the expression of E-cadherin increased following the transfection of A549 cells with a p300 small interfering RNA, potentially inhibiting mesenchymal transition and metastasis of LC (Chang et al., 2017). Hence, we concluded that SCFAs regulated the levels of acetylated histones by acting on both HDAC inhibitors and HAT activators. To translate this hypothesis into clinical practice, *in vivo* studies are needed for further evaluating the activity of isobutyrate and exploring the accurate mode of action of isobutyrate treatment in regulating GPCR expression and HAT activity.

This study has several limitations. First, the response of patients with LC to drugs varied depending on the pathological type and treatment strategy. The LC group comprised patients with both NSCLC and SCLC. Treatment strategies consisted of chemotherapy and targeted therapy. Therefore, differences between responders and non-responders might have been related to the pathological type and treatment strategies, as well as the length of PFS. However, we might partly confirm that our findings as the percentage of pathological types in each group were similar and fecal samples were collected before the initiation of treatment. Nevertheless, cohorts with larger and more homogeneous patients are needed to explore the distinct interactions between gut microbes and therapeutic efficacy in patients with LC. It might be beneficial to assess the differences between responders and non-responders to the same type of treatment within one broadly pathological type (NSCLC or SCLC) to exclude the confounding effects of pathological types and treatment strategies. In addition to the cancer type, the disease stage might also influence the composition of gut microbes and disease survival. Advanced diseases often respond worse to anti-cancer drugs; thus, we could not exclude that the observed microbial disturbances in patients with LC may be related to differences in cancer stage. In view of the fact that patients with advanced diseases more urgently need new targeted therapeutic approaches, it will be significant to compare responders and non-responders with metastatic LC in future research studies.

## Conclusion

In summary, we showed that the response to therapeutic drugs was related to specific alterations in the composition of gut microbes as well as serum concentrations of SCFAs in patients with LC. Moreover, *Parasutterella* and *Bacteroides_stercoris* are potential biomarkers for predicting the therapeutic efficacy in patients with LC. Isobutyrate effectively inhibited LC progression. These findings represent a key first step and emphasize the necessity to assess whether the gut microbes and SCFAs (especially isobutyrate) could be used as therapeutic targets in treating LC. Given the limited efficiency of current therapeutic strategies, we believe innovative anti-cancer strategies are urgently required.

## Data availability statement

The datasets presented in this study can be found in online repositories. The names of the repository/repositories and accession number(s) can be found below: https://www.ncbi.nlm.nih.gov/, SUB12510428. Further inquiries can be directed to the corresponding author/s.

## Ethics statement

The studies involving human participants were reviewed and approved by the Ethics Committee of Shengjing Hospital of China Medical University. The patients/participants provided their written informed consent to participate in this study.

## Author contributions

H-HC and T-NZ supervised and conceptualized the study. H-HC performed the most of experiments and was responsible for clinical sample collection and analyses, wrote the manuscript, and prepared Figures 1–5. Q-JW and Y-HZ are responsible for research supervision. Y-HZ is responsible for funding acquisition. All authors reviewed the manuscript.

## References

[B1] Adams G. N. Sharma B. K. Rosenfeldt L. Frederick M. Flick M. J. Witte D. P.. (2018). Protease-activated receptor-1 impedes prostate and intestinal tumor progression in mice. J. Thromb. Haemost. 16, 2258–2269. 10.1111/jth.14277 30152921 PMC6214773

[B2] Andrews M. C. Duong C. P. M. Gopalakrishnan V. Iebba V. Chen W. S. Derosa L.. (2021). Gut microbiota signatures are associated with toxicity to combined CTLA-4 and PD-1 blockade. Nat. Med. 27, 1432–1441. 10.1038/s41591-021-01406-6 34239137 PMC11107795

[B3] Anwar H. Iftikhar A. Muzaffar H. Almatroudi A. Allemailem K. S. Navaid S.. (2021). Biodiversity of gut microbiota: impact of various host and environmental factors. Biomed. Res. Int. 2021, 5575245. 10.1155/2021/5575245 34055983 PMC8133857

[B4] Bach Knudsen K. E. Laerke H. N. Hedemann M. S. Nielsen T. S. Ingerslev A. K. Gundelund Nielsen D. S.. (2018). Impact of diet-modulated butyrate production on intestinal barrier function and inflammation. Nutrients 10, 1499. 10.3390/nu10101499 30322146 PMC6213552

[B5] Becattini S. Taur Y. Pamer E. G. (2016). Antibiotic-induced changes in the intestinal microbiota and disease. Trends Mol. Med. 22, 458–478. 10.1016/j.molmed.2016.04.003 27178527 PMC4885777

[B6] Chang R. Zhang Y. Zhang P. Zhou Q. (2017). Snail acetylation by histone acetyltransferase p300 in lung cancer. Thorac. Cancer 8, 131–137. 10.1111/1759-7714.12408 28296173 PMC5415461

[B7] Gerard P. (2016). Gut microbiota and obesity. Cell Mol. Life Sci. 73, 147–162. 10.1007/s00018-015-2061-5 26459447 PMC11108539

[B8] Gopalakrishnan V. Helmink B. A. Spencer C. N. Reuben A. Wargo J. A. (2018). The influence of the gut microbiome on cancer, immunity, and cancer immunotherapy. Cancer Cell 33, 570–580. 10.1016/j.ccell.2018.03.015 29634945 PMC6529202

[B9] Hakozaki T. Richard C. Elkrief A. Hosomi Y. Benlaifaoui M. Mimpen I.. (2020). The gut microbiome associates with immune checkpoint inhibition outcomes in patients with advanced non-small cell lung cancer. Cancer Immunol. Res. 8, 1243–1250. 10.1158/2326-6066.CIR-20-0196 32847937

[B10] Heshiki Y. Vazquez-Uribe R. Li J. Ni Y. Quainoo S. Imamovic L.. (2020). Predictable modulation of cancer treatment outcomes by the gut microbiota. Microbiome 8, 28. 10.1186/s40168-020-00811-2 32138779 PMC7059390

[B11] Jin E. Wang W. Fang M. Wang L. Wu K. Zhang Y.. (2017). Lung cancer suppressor gene GPRC5A mediates p53 activity in nonsmall cell lung cancer cells in vitro. Mol. Med. Rep. 16, 6382–6388. 10.3892/mmr.2017.7343 28849235

[B12] Jin E. Wang W. Fang M. Wang W. Xie R. Zhou H.. (2019). Clinical significance of reduced GPRC5A expression in surgically resected non-small cell lung cancer. Oncol. Lett. 17, 502–507. 10.3892/ol.2018.9537 30655793 PMC6313189

[B13] Jin Y. Dong H. Xia L. Yang Y. Zhu Y. Shen Y.. (2019). The diversity of gut microbiome is associated with favorable responses to anti-programmed death 1 immunotherapy in chinese patients with NSCLC. J. Thorac. Oncol. 14, 1378–1389. 10.1016/j.jtho.2019.04.007 31026576

[B14] Kim K. Kwon O. Ryu T. Jung C. R. Kim J. Min J. K.. (2019). Propionate of a microbiota metabolite induces cell apoptosis and cell cycle arrest in lung cancer. Molec. Med. Rep. 20, 1569–1574. 10.3892/mmr.2019.10431 31257531 PMC6625448

[B15] Liu X. Cheng Y. Zang D. Zhang M. Li X. Liu D.. (2021a). The role of gut microbiota in lung cancer: from carcinogenesis to immunotherapy. Front. Oncol. 11, 720842. 10.3389/fonc.2021.720842 34490119 PMC8417127

[B16] Liu X. Pan X. Liu H. Ma X. (2021b). Gut microbial diversity in female patients with invasive mole and choriocarcinoma and its differences versus healthy controls. Front. Cell Infect. Microbiol. 11, 704100. 10.3389/fcimb.2021.704100 34513727 PMC8428518

[B17] Liu X. Yu J. Song S. Yue X. Li Q. (2017). Protease-activated receptor-1 (PAR-1): a promising molecular target for cancer. Oncotarget 8, 107334–107345. 10.18632/oncotarget.21015 29291033 PMC5739818

[B18] Lozupone C. A. Stombaugh J. I. Gordon J. I. Jansson J. K. Knight R. (2012). Diversity, stability and resilience of the human gut microbiota. Nature 489, 220–230. 10.1038/nature11550 22972295 PMC3577372

[B19] Mancabelli L. Milani C. Lugli G. A. Turroni F. Cocconi D. van Sinderen D.. (2017). Identification of universal gut microbial biomarkers of common human intestinal diseases by meta-analysis. FEMS Microbiol. Ecol. 93, 153. 10.1093/femsec/fix153 29126267

[B20] Marques C. Oliveira C. S. Alves S. Chaves S. R. Coutinho O. P. Corte-Real M.. (2013). Acetate-induced apoptosis in colorectal carcinoma cells involves lysosomal membrane permeabilization and cathepsin D release. Cell Death Dis. 4, e507. 10.1038/cddis.2013.29 23429293 PMC3734821

[B21] Martinez C. Gonzalez-Castro A. Vicario M. Santos J. (2012). Cellular and molecular basis of intestinal barrier dysfunction in the irritable bowel syndrome. Gut. Liver 6, 305–315. 10.5009/gnl.2012.6.3.305 22844557 PMC3404166

[B22] Messaoudene M. Pidgeon R. Richard C. Ponce M. Diop K. Benlaifaoui M.. (2022). A natural polyphenol exerts antitumor activity and circumvents anti-PD-1 resistance through effects on the gut microbiota. Cancer Discov. 12, 1070–1087. 10.1158/2159-8290.CD-21-0808 35031549 PMC9394387

[B23] Miao J. Fan Q. Cui L. Li X. Wang H. Ning G.. (2010). The MYST family histone acetyltransferase regulates gene expression and cell cycle in malaria parasite Plasmodium falciparum. Mol. Microbiol. 78, 883–902. 10.1111/j.1365-2958.2010.07371.x 20807207 PMC2978264

[B24] Mirzaei R. Afaghi A. Babakhani S. Sohrabi M. R. Hosseini-Fard S. R. Babolhavaeji K.. (2021). Role of microbiota-derived short-chain fattyacids in cancer development and prevention. Biomed. Pharmacother 139, 111619. 10.1016/j.biopha.2021.111619 33906079

[B25] Murugesan S. Nirmalkar K. Hoyo-Vadillo C. Garcia-Espitia M. Ramirez-Sanchez D. Garcia-Mena J. (2018). Gut microbiome production of short-chain fatty acids and obesity in children. Eur. J. Clin. Microbiol. Infect. Dis. 37, 621–625. 10.1007/s10096-017-3143-0 29196878

[B26] Nomura M. Nagatomo R. Doi K. Shimizu J. Baba K. Saito T.. (2020). Association of short-chain fatty acids in the gut microbiome with clinical response to treatment with nivolumab or pembrolizumab in patients with solid cancer tumors. JAMA Netw. Open 3, e202895. 10.1001/jamanetworkopen.2020.2895 32297948 PMC7163404

[B27] Pi H. Huang L. Liu H. Liang S. Mei J. (2020). Effects of PD-1/PD-L1 signaling pathway on intestinal flora in patients with colorectal cancer. Cancer Biomark 28, 529–535. 10.3233/CBM-201606 32568184 PMC12662382

[B28] Ribelli G. Simonetti S. Iuliani M. Rossi E. Vincenzi B. Tonini G.. (2021). Osteoblasts promote prostate cancer cell proliferation through androgen receptor independent mechanisms. Front. Oncol. 11, 789885. 10.3389/fonc.2021.789885 34966687 PMC8711264

[B29] Rinninella E. Cintoni M. Raoul P. Lopetuso L. R. Scaldaferri F. Pulcini G.. (2019). Food components and dietary habits: keys for a healthy gut microbiota composition. Nutrients 11, 2393. 10.3390/nu11102393 31591348 PMC6835969

[B30] Sawada Y. Kikugawa T. Iio H. Sakakibara I. Yoshida S. Ikedo A.. (2020). GPRC5A facilitates cell proliferation through cell cycle regulation and correlates with bone metastasis in prostate cancer. Int. J. Cancer 146, 1369–1382. 10.1002/ijc.32554 31276604

[B31] Siegel R. L. Miller K. D. Jemal A. (2019). Cancer statistics, 2019. CA Cancer J. Clin. 69, 7–34. 10.3322/caac.21551 30620402

[B32] Singh N. Gurav A. Sivaprakasam S. Brady E. Padia R. Shi H.. (2014). Activation of Gpr109a, receptor for niacin and the commensal metabolite butyrate, suppresses colonic inflammation and carcinogenesis. Immunity 40, 128–139. 10.1016/j.immuni.2013.12.007 24412617 PMC4305274

[B33] Tang Y. Chen Y. Jiang H. Robbins G. T. Nie D. (2011). G-protein-coupled receptor for short-chain fatty acids suppresses colon cancer. Int. J. Cancer 128, 847–856. 10.1002/ijc.25638 20979106

[B34] Tremaroli V. Backhed F. (2012). Functional interactions between the gut microbiota and host metabolism. Nature 489, 242–249. 10.1038/nature11552 22972297

[B35] Vernocchi P. Gili T. Conte F. Del Chierico F. Conta G. Miccheli A.. (2020). Network analysis of gut microbiome and metabolome to discover microbiota-linked biomarkers in patients affected by non-small cell lung cancer. Int. J. Mol. Sci. 21, 8730. 10.3390/ijms21228730 33227982 PMC7699235

[B36] Wang T. Cai G. Qiu Y. Fei N. Zhang M. Pang X.. (2012). Structural segregation of gut microbiota between colorectal cancer patients and healthy volunteers. ISME J. 6, 320–329. 10.1038/ismej.2011.109 21850056 PMC3260502

[B37] West N. R. Powrie F. (2015). Immunotherapy not working? Check your microbiota. Cancer Cell 28, 687–689. 10.1016/j.ccell.2015.11.010 26678336

[B38] Wu J. Zhang C. Xu S. Xiang C. Wang R. Yang D.. (2020). Fecal microbiome alteration may be a potential marker for gastric cancer. Dis. Markers 2020, 3461315. 10.1155/2020/3461315 33014185 PMC7519184

[B39] Wu J. Zhou Z. Hu Y. Dong S. (2012). Butyrate-induced GPR41 activation inhibits histone acetylation and cell growth. J. Genet. Genom. 39, 375–384. 10.1016/j.jgg.2012.05.008 22884094

[B40] Wu X. Wu Y. He L. Wu L. Wang X. Liu Z. (2018). Effects of the intestinal microbial metabolite butyrate on the development of colorectal cancer. J. Cancer 9, 2510–2517. 10.7150/jca.25324 30026849 PMC6036887

[B41] Wu Z. Zeng Y. Zhong M. Wang B. (2014). Targeting A549 lung adenocarcinoma cell growth and invasion with proteaseactivated receptor1 siRNA. Mol. Med. Rep. 9, 1787–1793. 10.3892/mmr.2014.2023 24604247

[B42] Xia Y. Zhang X. L. Jin F. Wang Q. X. Xiao R. Hao Z. H.. (2016). Apoptotic effect of sodium acetate on a human gastric adenocarcinoma epithelial cell line. Genet. Mol. Res 15, 375. 10.4238/gmr.15048375 27808354

[B43] Yu D. C. Bury J. P. Tiernan J. Waby J. S. Staton C. A. Corfe B. M. (2011). Short-chain fatty acid level and field cancerization show opposing associations with enteroendocrine cell number and neuropilin expression in patients with colorectal adenoma. Mol. Cancer 10, 27. 10.1186/1476-4598-10-27 21401950 PMC3068125

[B44] Zhang H. Wang L. Bai J. Jiao W. Wang M. (2020). MIER3 suppresses the progression of non-small cell lung cancer by inhibiting Wnt/beta-Catenin pathway and histone acetyltransferase activity. Transl. Cancer Res. 9, 346–357. 10.21037/tcr.2020.01.07 35117188 PMC8798777

[B45] Zhao L. Bin S. He H.-L. Yang J.-M. Pu Y.-C. Gao C.-H.. (2018). Sodium butyrate increases P-gp expression in lung cancer by upregulation of STAT3 and mRNA stabilization of ABCB1. Anti-Cancer Drugs. 29, 227–233. 10.1097/CAD.0000000000000588 29293118

