# Supplementary material for: Gut microbiome and serum short-chain fatty acids are associated with responses to chemo- or targeted therapies in Chinese patients with lung cancer
Source: Front Microbiol. 2023 Jul 19;14:1165360. doi: 10.3389/fmicb.2023.1165360 (PMC10411610; doi:10.3389/fmicb.2023.1165360)
Supplement: Supplementary file 1 [file Data_Sheet_1.docx]

**Supplementary material 1：Method Detail**

**Study population and samples collection**

*Study population*

Patients with lung cancer were recruited at the Shengjing Hospital of China Medical University between June and December 2020. Patients were included at the time of diagnosis and before the start of systemic therapy. Patients provided written informed consent to participate in the study, which was approved by the Ethics Committee of Shengjing Hospital of China Medical University (no. 2020PS556K). Inclusion criteria were as follows: 1) patients older than 30 years and younger than 75 years; 2) patients with a recent diagnosis of lung cancer based on pathology; and 3) patients who received chemotherapy or targeted therapy. Exclusion criteria were as follows: 1) patients with lung cancer without a pathological diagnosis or diagnosed with nonprimary lung cancer; 2) patients who received radiotherapy or immunotherapy; and 3) patients who refused to cooperate with providing serum and stool samples.

Disease severity was evaluated by computed tomography or magnetic resonance imaging, while therapeutic response was evaluated using Response Evaluation Criteria in Solid Tumors 1.1 (RECIST 1.1). Clinical response to treatment with systemic therapy was evaluated every 8 weeks and was confirmed by a subsequent assessment no less than 4 weeks thereafter. Responsive and nonresponsive patients were defined based on the following criteria (Hodi et al. 2018): patients with durable clinical benefits (defined as no progression event or death within the first 6 months of systemic therapy) were classified as responders, whereas those presenting no durable clinical benefits (progression event or death within the first 6 months of systemic therapy) were classified as non-responders.

*Collection of clinical data, serum, and stool samples*

Clinical information concerning sex, age, clinical stage, smoking status, pathological type, and Eastern Cooperative Oncology Group (ECOG) scores were collected using a questionnaire. In addition, blood samples were collected in blood collection tubes containing ethylenediaminetetraacetic acid (EDTA) after patients fasted for 8 h. Blood samples (2 mL per patient) were stored in a refrigerator at 4 °C. Blood samples were centrifuged at 3500 rpm for 10 min. After that, the supernatant was transferred to a 1 mL strain preservation tube and then stored at –80 °C until further analysis. Stool samples were collected at a time point prior to treatment. Participants received a plastic container and stool collection device to ensure hygienic sampling. Samples were aliquoted into 2 mL sterile tubes and stored at –80 °C until further analysis.

**16S ribosomal DNA (rDNA) gene sequencing**

*Extraction of genome DNA:* Total genomic DNA was extracted using the cetyltrimethylammonium bromide (CTAB) method. DNA concentration and purity were monitored on a 1% agarose gel. According to the concentration, DNA was diluted to 1 ng/μL using sterile water.

*Amplicon Generation:* 16S rDNA genes were amplified using a specific primer with the barcode (16S V3-V4: 341F CCTAYGGGRBGCASCAG; 806RGGACTACNNGGGTATCTAAT). All polymerase chain reactions (PCR) were carried out in 30 μL reactions with 15 μL Phusion High-Fidelity PCR Master Mix (New England Biolabs), 0.2 μM forward and reverse primers, and approximately 10 ng template DNA. Thermal cycling consisted of initial denaturation at 98 °C for 1 min, followed by 30 cycles of denaturation at 98 °C for 10 s, annealing at 50 °C for 30 s, and elongation at 72 °C for 30 s. Finally, samples were kept on hold at 72 °C for 5 min.

*Quantification and quantitation of PCR products:* The same volume of 1× loading buffer (containing SYBR green) was mixed with PCR products and electrophoresed on an 2% agarose gel for detection. Samples with a bright main strip between 400–450 bp were chosen for further experiments.

*Mixing and purification of PCR products:* PCR products were mixed at equidensity ratios. Then, PCR products were purified using the GeneJET Gel Extraction Kit (Thermo Scientific).

*Library preparation and sequencing:* Sequencing libraries were generated using the Illumina TruSeq DNA PCR-Free Library Preparation Kit (Illumina, USA) following the manufacturer’s recommendations, and index codes were added. Library quality was assessed on a Qubit 2.0 Fluorometer (Thermo Scientific) and Agilent Bioanalyzer 2100 system (Agilent). Finally, the library was sequenced on an Illumina NovaSeq platform and 250 bp paired-end reads were generated.

**Analysis of concentration of** **SCFAs**

*Chemicals and reagents:* Methyl tert-butyl ether (MTBE) was purchased from CNW (CNW Technologies, Germany). Milli-Q water (Millipore, Bradford, USA) was used for all experiments. All standards were purchased from CNW (Beijing, China) or Aladdin (Shanghai, China). Stock solutions of standards were prepared in MTBE at a concentration of 1 mg/mL. All stock solutions were stored at –20 °C. Stock solutions were diluted with MTBE to obtain working solutions before analysis.

*Sample preparation and extraction:* Serum samples were thawed and vortexed for 1 min prior to analysis. Briefly, 50 μL of samples were added to a 1.5 mL tube and 100 μL phosphoric acid (0.5% v/v) solution was added to the tube and the mixture was vortexed for 3 min. Next, 150 μL MTBE (containing an internal standard) solution was added. The mixture was vortexed for 3 min and then ultrasonicated for 5 min. After that, the mixture was centrifuged at 12 000 r/min for 10 min at 4 °C. The supernatant was collected and used for gas chromatography-tandem mass spectrometry (GC-MS/MS) analysis (Zhao et al. 2006).

*GC-MS/MS analysis:* An Agilent 7890 B gas chromatograph coupled to a 7000D mass spectrometer with a DB-FFAP column (30 m length × 0.25 mm i.d. × 0.25 μm film thickness, J&W Scientific, USA) was used for GC-MS/MS analysis of SCFAs. Helium was used as the carrier gas at a flow rate of 1.2 mL/min. Injection was performed in split mode, at a volume of 2 μL. The oven temperature was held at 90 °C for 1 min, raised to 100 °C at a rate of 25 °C/min, then raised to 150 °C at a rate of 20 °C/min, held there for 0.6 min, raised to 200 °C at a rate of 25 °C/min, held there for 0.5 min, after running for 3 min. All samples were analyzed in the multiple reaction monitoring mode. The injector inlet and transfer line temperatures were 200 °C and 230 °C, respectively (Zhao, Nyman and Jonsson 2006, Zhao et al. 2017).

**Statistical analysis**

*Statistical analysis of gut microbiota data*

Paired-end reads from the original DNA fragments were merged using FLASH (Magoc and Salzberg 2011), a very fast and accurate analysis tool designed to merge paired-end reads when there are overlaps between reads1 and reads2. Paired-end reads were assigned to each sample according to their unique barcodes. Sequences were analyzed using the QIIME (Caporaso et al. 2010) software package (Quantitative Insights Into Microbial Ecology), and in-house Perl scripts were used to analyze α-(within samples) and β-(among samples) diversity. First, reads were filtered using the QIIME quality filters. Then, we used pick_de_novo_otus.py to pick operational taxonomic units (OTUs) by making an OTU Table. Sequences with ≥ 97 % similarity were assigned to the same OTUs. We selected representative sequences for each OTU and used the Ribosomal Database Project (RDP) classifier (Wang et al. 2007) to annotate the taxonomic information for each representative sequence. To compute α-diversity, we rarified the OTU Table and calculated 3 metrics: Chao1 that estimates species abundance, Shannon that estimates species evenness, and Simpson that estimates species richness. Rarefaction curves were generated based on these metrics.

QIIME calculates the bray_curtis, which is a phylogenetic measure of β-diversity. bray_curtis was used for principal coordinate analysis (PCoA). PCoA helps obtain and visualize principal coordinates from complex multidimensional data by transforming them from a distance matrix to a new set of orthogonal axes. Briefly, the maximum variation factor is demonstrated by the first principal coordinate, the second maximum by the second principal coordinate, and so on. To mine deeper data on microbial diversity between samples, significance tests were conducted using certain statistical analysis methods, including *t*-test, MetaStat, and LEfSe.

*Statistical analysis of SCFA levels*

Statistical analyses were performed using R, version 4.0.0. To assess potential differences in the distribution of responders versus non-responders, a *t*-test was performed. Statistical significance was set at *p* < 0.05.

*Spearman correlation analysis*

Spearman correlation analysis was used to calculate the correlation between metabolites. All data were calculated using the cor function in R version 4.0.0. The correlation data between SCFAs and differential microorganisms were extracted and a heatmap was drawn using the corrplot package in R**.** The significance of correlation was calculated using the corPvalueStudent function of the weighted gene co-expression network analysis (WGCNA) package in R. Statistical significance was set at *p* < 0.05.

*Statistical analysis of in vitro experiments*

Results are expressed as the mean ± standard deviation (SD) of 3 independent experiments. The Student's *t*-test in Microsoft Excel 2019 (Microsoft Corporation) was used to assess significance. Statistical significance was set at *p* < 0.05.

**References**

1 Hodi, F. S., [Ballinger](https://pubmed.ncbi.nlm.nih.gov/?size=200&term=Ballinger+M&cauthor_id=29341833), M., [Lyons](https://pubmed.ncbi.nlm.nih.gov/?size=200&term=Lyons+B&cauthor_id=29341833), B., [Soria](https://pubmed.ncbi.nlm.nih.gov/?size=200&term=Soria+JC&cauthor_id=29341833), J-C., [Nishino](https://pubmed.ncbi.nlm.nih.gov/?size=200&term=Nishino+M&cauthor_id=29341833), M., [Tabernero](https://pubmed.ncbi.nlm.nih.gov/?size=200&term=Tabernero+J&cauthor_id=29341833), J. *et al.* (2018) Immune-Modified Response Evaluation Criteria In Solid Tumors (imRECIST): Refining Guidelines to Assess the Clinical Benefit of Cancer Immunotherapy. *J Clin Oncol* 36, 850-858, doi:10.1200/JCO.2017.75.1644.

2 Zhao, G., Nyman, M. and Jonsson, J. A. (2006) Rapid determination of short-chain fatty acids in colonic contents and faeces of humans and rats by acidified water-extraction and direct-injection gas chromatography. *Biomed Chromatogr* 20, 674-682, doi:10.1002/bmc.580.

3 Zhao, R., [Chu](https://pubmed.ncbi.nlm.nih.gov/?size=200&term=Chu+L&cauthor_id=28237548), L., [Wang](https://pubmed.ncbi.nlm.nih.gov/?size=200&term=Wang+Y&cauthor_id=28237548), Y., [Song](https://pubmed.ncbi.nlm.nih.gov/?size=200&term=Song+Y&cauthor_id=28237548), Y., [Liu](https://pubmed.ncbi.nlm.nih.gov/?size=200&term=Liu+P&cauthor_id=28237548), P., [Li](https://pubmed.ncbi.nlm.nih.gov/?size=200&term=Li+C&cauthor_id=28237548), C., *et al.* (2017) Application of packed-fiber solid-phase extraction coupled with GC-MS for the determination of short-chain fatty acids in children's urine. *Clin Chim Acta* 468, 120-125, doi:10.1016/j.cca.2017.02.016.

4 Magoc, T. and Salzberg, S. L. (2011) FLASH: fast length adjustment of short reads to improve genome assemblies. *Bioinformatics* 27, 2957-2963, doi:10.1093/bioinformatics/btr507.

5 Caporaso, J. G., [Kuczynski](https://pubmed.ncbi.nlm.nih.gov/?size=200&term=Kuczynski+J&cauthor_id=20383131), J., [Stombaugh](https://pubmed.ncbi.nlm.nih.gov/?size=200&term=Stombaugh+J&cauthor_id=20383131), J., [Bittinger](https://pubmed.ncbi.nlm.nih.gov/?size=200&term=Bittinger+K&cauthor_id=20383131), K., [Bushman](https://pubmed.ncbi.nlm.nih.gov/?size=200&term=Bushman+FD&cauthor_id=20383131), F.D., [Costello](https://pubmed.ncbi.nlm.nih.gov/?size=200&term=Costello+EK&cauthor_id=20383131), E.K., *et al.* (2010) QIIME allows analysis of high-throughput community sequencing data. *Nat Methods* **7**, 335-336, doi:10.1038/nmeth.f.303.

6 Wang, Q., Garrity, G. M., Tiedje, J. M. and Cole, J. R. (2007) Naive Bayesian classifier for rapid assignment of rRNA sequences into the new bacterial taxonomy. *Appl Environ Microbiol* 73, 5261-5267, doi:10.1128/AEM.00062-07.
