# Supplementary material for: Gut microbiome and serum short-chain fatty acids are associated with responses to chemo- or targeted therapies in Chinese patients with lung cancer
Source: Front Microbiol. 2023 Jul 19;14:1165360. doi: 10.3389/fmicb.2023.1165360 (PMC10411610; doi:10.3389/fmicb.2023.1165360)
Supplement: Supplementary file 2 [file Data_Sheet_2.PDF]

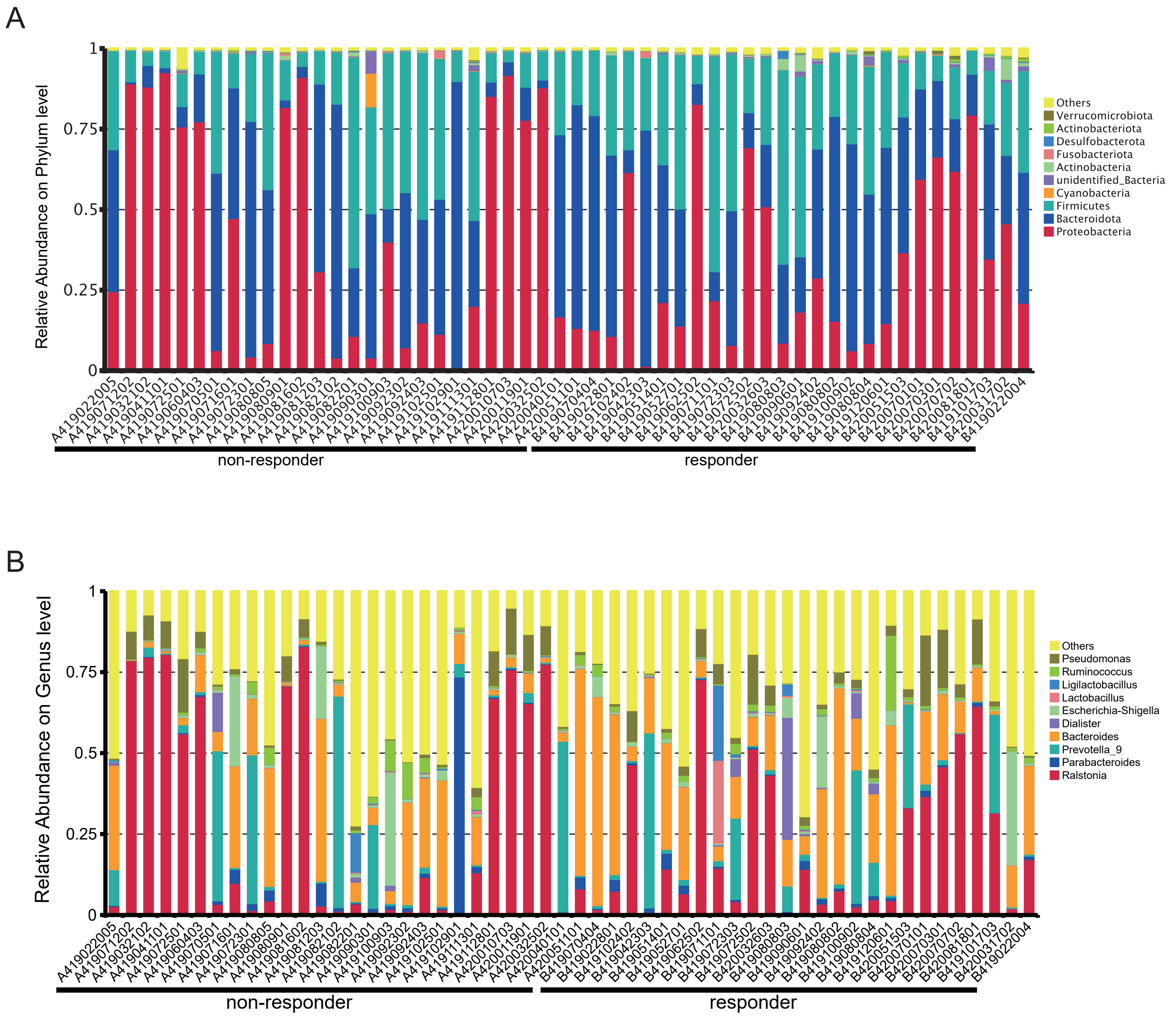

Supplementary Figure 1: The taxonomic composition distribution in responders (n=26) and non-responders (n=28). (A) Relative abundance of bacterial phyla in microbiota. (B) Relative abundance of bacterial genus in microbiota.
