# Supplementary material for: Gut microbiome and serum short-chain fatty acids are associated with responses to chemo- or targeted therapies in Chinese patients with lung cancer
Source: Front Microbiol. 2023 Jul 19;14:1165360. doi: 10.3389/fmicb.2023.1165360 (PMC10411610; doi:10.3389/fmicb.2023.1165360)
Supplement: Supplementary file 3 [file Data_Sheet_3.PDF]

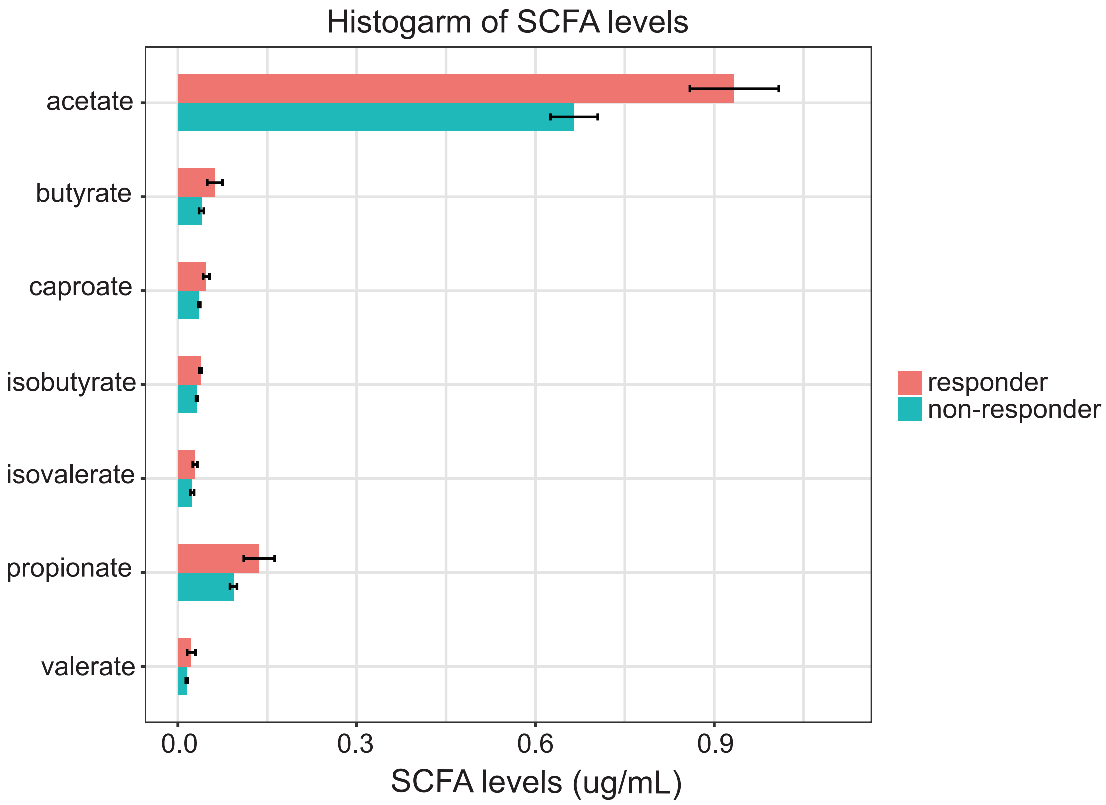

Supplementary Figure 2: Histogarm of serum levels of acetate, butyrate, caproate, isobutyrate, isovalerate, propionate and valerate separately. Each of the short-chain fatty acids (SCFA) was found to be reduced in non-responders (n = 30) compared with responders (n = 30).
