# Supplementary material for: Gut microbiome and serum short-chain fatty acids are associated with responses to chemo- or targeted therapies in Chinese patients with lung cancer
Source: Front Microbiol. 2023 Jul 19;14:1165360. doi: 10.3389/fmicb.2023.1165360 (PMC10411610; doi:10.3389/fmicb.2023.1165360)
Supplement: Supplementary file 4 [file Table_1.DOCX]

**Supplementary Table 1: Primers for GPCRs expression identification in lung cancer cell lines.**

| NO. | Primer names | Sequences |
| --- | --- | --- |
| 1 | GPR41 F | TGGTTCGTCTTCTCGGTGTA |
|  | GPR41 R | CGGTCAGGTTGAGCAGGA |
| 2 | GPR43 F | CTACCTGGGAGTGGCTTTC |
|  | GPR43 R | TGACCTGCTCAGTCGTGTT |
| 3 | GPRC5A F | ATGGCTACAACAGTCCCT |
|  | GPRC5A R | CCAAAGATGCCCAACAC |
| 4 | PAR1 F | GCCATTACTCTGAAGTCCTAC |
|  | PAR1 R | CTACCTGTGATGCCGAAC |
| 5 | β-actin F | GGCACCCAGCACAATGAA |
|  | β-actin R | TAGAAGCATTTGCGGTGG |

GPCR: G protein coupled receptors; F: Forward; R: Reverse
