# Supplementary material for: Gut microbiome and serum short-chain fatty acids are associated with responses to chemo- or targeted therapies in Chinese patients with lung cancer
Source: Front Microbiol. 2023 Jul 19;14:1165360. doi: 10.3389/fmicb.2023.1165360 (PMC10411610; doi:10.3389/fmicb.2023.1165360)
Supplement: Supplementary file 5 [file Table_2.DOCX]

| NO. | Biomarker names | Logarithm value | Groups | LDA value | P value |
| --- | --- | --- | --- | --- | --- |
| 1 | s_Bacteroides_stercoris | 4.53484 | NR | 4.070256 | 0.004284 |
| 2 | s_Clostridiaceae_bacterium_DJF_VR76 | 4.001247 | NR | 3.775326 | 2.43E-05 |
| 3 | g_Christensenellaceae_R_7_group | 4.046012 | NR | 3.747014 | 0.00047 |
| 4 | f_Christensenellaceae | 4.046566 | NR | 3.746854 | 0.000691 |
| 5 | o_Christensenellales | 4.046566 | NR | 3.746854 | 0.000691 |
| 6 | f_Oscillospiraceae | 4.100568 | NR | 3.556092 | 0.010933 |
| 7 | c_unidentified_Actinobacteria | 3.949311 | NR | 3.519728 | 0.036191 |
| 8 | p_Actinobacteria | 3.949311 | NR | 3.519728 | 0.036191 |
| 9 | o_Bifidobacteriales | 3.885953 | NR | 3.516048 | 0.002744 |
| 10 | f_Bifidobacteriaceae | 3.885953 | NR | 3.516048 | 0.002744 |
| 11 | g_Bifidobacterium | 3.885553 | NR | 3.51603 | 0.00325 |
| 12 | f_Rikenellaceae | 4.07556 | NR | 3.422201 | 0.014648 |
| 13 | g_UCG_002 | 3.783037 | NR | 3.323837 | 0.031816 |
| 14 | s_Bacteroides_coprophilus | 3.70003 | NR | 3.322183 | 0.002787 |
| 15 | g_Eubacterium__ruminantium_group | 3.781983 | NR | 3.278201 | 9.81E-05 |
| 16 | p_unidentified_Bacteria | 3.83493 | NR | 3.251233 | 0.03182 |
| 17 | f_Enterococcaceae | 3.494049 | NR | 3.156539 | 0.002303 |
| 18 | g_Enterococcus | 3.494049 | NR | 3.156539 | 0.002303 |
| 19 | s_Enterococcus_faecium | 3.488237 | NR | 3.151367 | 0.00362 |
| 20 | o_Acidaminococcales | 3.587993 | NR | 3.137861 | 0.021308 |
| 21 | f_Acidaminococcaceae | 3.587993 | NR | 3.137861 | 0.021308 |
| 22 | s_Bifidobacterium_breve | 3.372768 | NR | 3.134822 | 0.000202 |
| 23 | s_Lactobacillus_aviarius | 3.378445 | NR | 3.122404 | 0.002682 |
| 24 | o_Peptostreptococcales_Tissierellales | 3.859827 | NR | 3.057708 | 0.022303 |
| 25 | s_Bacteroides_clarus | 3.679231 | NR | 3.045737 | 0.02279 |
| 26 | c_Alphaproteobacteria | 3.259192 | NR | 3.005539 | 7.49E-05 |
| 27 | s_Haemophilus_parainfluenzae | 3.429571 | R | 3.034543 | 4.00E-05 |
| 28 | g_Citrobacter | 3.498432 | R | 3.036008 | 0.003632 |
| 29 | g_Haemophilus | 3.455492 | R | 3.04485 | 7.28E-05 |
| 30 | f_Pasteurellaceae | 3.48395 | R | 3.06156 | 9.71E-05 |
| 31 | g_Prevotella_7 | 3.492339 | R | 3.207453 | 4.44E-06 |
| 32 | f_unidentified_Gastranaerophilales | 3.589983 | R | 3.339725 | 0.000941 |
| 33 | g_unidentified_Gastranaerophilales | 3.589983 | R | 3.339733 | 0.000941 |
| 34 | s_Candidatus_Melainabacteria_bacterium_MEL_A1 | 3.589782 | R | 3.33996 | 6.35E-05 |
| 35 | s_Bacteroides_dorei | 3.849671 | R | 3.466355 | 0.000104 |
| 36 | o_Clostridiales | 4.009739 | R | 3.687128 | 0.026697 |
| 37 | f_Clostridiaceae | 4.009632 | R | 3.687364 | 0.025534 |
| 38 | g_Clostridium_sensu_stricto_1 | 4.007768 | R | 3.695353 | 0.01396 |
| 39 | k_Bacteria | 5.99994 | R | 3.766994 | 0.009382 |

**Supplementary Table 2. Characteristics of differential microorganisms of LEfSe analysis (LDA > 3).**

R: responder; NR: non-responder
